# Good long-term patients reported outcomes, return-to-work and return-to-sport rate and survivorship after posterior cruciate ligament (PCL)-based multiligament knee injuries (MLKI) with posteromedial corner tears as significant risk factor for failure

**DOI:** 10.1007/s00167-023-07547-0

**Published:** 2023-09-05

**Authors:** Nicola Pizza, Stefano Di Paolo, Alberto Grassi, Anna Pagano, Marianna Viotto, Giacomo Dal Fabbro, Piero Agostinone, Gian Andrea Lucidi, Juan Carlos Monllau, Stefano Zaffagnini

**Affiliations:** 1 Knee and Arthroscopy Unit, ICATME, Hospital Universitari Dexeus, Universitat Autònoma de Barcelona, Barcelona, Spain; 2 Clinica Ortopedica e Traumatologica II, IRCCS Istituto Ortopedico Rizzoli, Bologna, Italy; 3 Dipartimento di Scienze Biomediche e Neuromotorie, DIBINEM, Università di Bologna, Bologna, Italy

**Keywords:** PCL-based MLKI, PMC, PROMS, Failure, Survivorship

## Abstract

**Purpose:**

To assess the survival rate and associated risk factors of a wide cohort of patient’s underwent surgical treatment for posterior cruciate ligament (PCL)-based multiligament knee injury (MLKI) at long-term follow-up and to investigate the long-term patient’s reported outcomes (PROMS) and functional activity.

**Methods:**

All cases of PCL-based MLKI performed at one single sport-medicine institution were extracted and patient’s with a minimum 2 years of follow-up included. VAS, Lysholm, KOOS, Tegner Activity level scores, the incidence and time of return to sport (RTS) and return to work (RTW) were collected before, after surgery and at final follow-up. A multivariate logistic regression was performed to investigate the outcomes associated with the patient’s acceptable symptoms state (PASS) for each sub-score of the KOOS. The Kaplan–Meier method with surgical failure (re-operation to one of the reconstructed ligaments) as endpoint was used to perform the survivorship analysis for the entire cohort.

**Results:**

Forty-two patients were included and evaluated at an average of 10 years. All PROMS significantly improved from pre- to post-surgery (range η_p_^2^ 0.21–0.43, *p* < 0.05) except for the Tegner score which significantly improved from pre-surgery and to final follow-up (η_p_^2^ = 0.67, *p* < 0.001). RTW was achieved in the 95.2% after 2.4 ± 1.9 months. RTS was achieved in 78.6% after 6.7 ± 5.0 months. The higher number of surgeries were the significant negative predictors of PASS for the KOOS sub-scales Sport (*p* = 0.040) and Quality of Life (*p* = 0.046), while the presence of meniscal lesions was a significant negative predictor of PASS only for the KOOS sub-scale of Sport (*p* = 0.003). Six patients (14.3%) underwent reoperation and were considered as surgical failures. The global survivorship was 95.2%, 92.6%, 87.1%, and 74.7% at 2, 5, 12, and 15 years, respectively. The survivorship in patient undergoing PMC reconstruction surgery was significantly lower (*p* = 0.004; HR 7.1) compared to patients without a PMC lesion.

**Conclusion:**

Good-to-excellent PROMS could be obtained and maintained at long-term follow-up after surgery, with the higher number of surgeries and meniscal lesions as significant negative predictors of the PASS. Moreover, the presence of a PMC lesion significantly increases the risk of the PCL reconstruction failure.

**Level of evidence:**

III.

## Introduction

Multiple ligament knee injuries (MLKI) are as rare as possibly devastating condition. Even though account for only 0.02–0.2% of all orthopedic injuries the real incidence could be overlooked due to the subtle diagnosis and the occurrence of autonomously reduced knee dislocation [17, 22]. MLKI are defined as the lesion of at least two of the four main ligamentous structures of the knee, and its classification follows the one proposed by Schenk et al. [24] According to Fanelli, the isolated lesions of the posterior cruciate ligament (PCL) are rare respect to its association with other knee ligaments injuries defining the so-called PCL-based multiligament knee injuries which account for the 96% of cases [5]. The association of structures involved is extremely variable due to injury mechanism, patient’s characteristics, and knee anatomy itself [2, 6, 10] and this account for many different scenarios to be managed by the surgeon. Of these, bi-cruciate (Anterior Cruciate Ligament—ACL; Posterior Cruciate Ligament—PCL) injuries are often associated with medial and/or lateral structures involvement representing one of the most severe conditions. So far, the best solution to improve clinical outcomes is represented by the surgical intervention of the torn ligaments. However, several issues are still present regarding different topics such as the timing of surgery, the use of allograft or autograft, the number of procedures needed, if repair or reconstruct and which ligament reconstruct first. Another crucial topic to be investigated is the likelihood for patients to undergo further surgical procedures and which are the correlated risk factors. Moreover, if good outcomes have been reported in the short-term there is a lack of studies investigating the outcomes of such procedures in the long-term.

Hence, the purpose of the present study was to assess the survival rate and associated risk factors of a wide cohort of patient’s underwent surgical treatment for PCL-based MLKI at long-term follow-up and to investigate the long-term patient’s reported outcomes (PROMS) and functional activity.

The clinical relevance of this study derives from the deepening of the knowledge into the MLKI which can help the surgeon to set patient’s expectations in these complex scenarios.

## Material and methods

All procedures described in this study were performed in accordance with the ethical standards of the 1964 Helsinki declaration and its later amendments and Rizzoli Orthopedic Institute Ethic Committee approval was obtained (Internal Protocol CE AVEC: 147/2021/Oss/IOR). All patients signed an informed consent form before recruitment. No external funding was received for the initiation or completion of this study.

The institutional database was retrospectively searched for patients who underwent isolated and/or combined PCL reconstruction between January 2000 and December 2019.

Medical charts were reviewed to collect patients’ information, anamnesis, and surgical data. All the patients were contacted to assess the occurrence of further surgical procedures.

The VAS, Lysholm, KOOS, and Tegner Activity level scores were collected for each patient before, after surgery and at final follow-up. The incidence and time of return to sport (RTS) and return to work (RTW) were collected.

### Statistical analysis

The sample size was determined according to an a-priori power analysis in G*Power (v3.1, Brunsbüttel, Germany). The calculation was based on a repeated measure ANOVA considering the three time frames (pre-surgery, post-surgery, final follow-up) for PROMS. A within group variance of 20 points in PROMS (Lysholm score) emerged from literature [12, 14]. Hypothesizing a conservative variance explained by the time effect of 1 point, an effect size of 0.22 emerged. Considering an alfa = 0.05 and a power of 0.8, the minimum sample size required was identified in 34 patients.

Descriptive statistics (mean, standard deviation, confidence intervals) were collected for each score at each time frame. The ANOVA was used to investigate the improvement in PROMS after surgery. A multivariate logistic regression was performed to investigate the outcomes associated with the patient’s acceptable symptoms state (PASS) for each sub-score of the KOOS.

Survival analysis was performed via the Kaplan–Meier method with surgical failure (re-operation to one of the reconstructed ligaments) as endpoint. The mean estimated survival time was calculated for the entire cohort, furthermore, the survivorship of ACL-PCL-PMC, ACL-PCL-PLC, and if a PMC lesion was present, were compared. The log-rank test with hazard ratio was calculated, with *p* < 0.05 set as level of statistical significance. A logistic regression was also performed by using sex, BMI (≤ 25 vs > 25), age at surgery, limb, presence of cartilage lesions, diabetes, smoke, meniscal lesion as independent variables.

## Results

A total of 42 consecutive patients (39 males and 3 females) were included and evaluated at an average of 10 years with minimum 2 years follow-up (Table 1). 11 patients underwent isolated PCL-reconstruction (26%), 2 ACL + PCL (4.7%), 9 PCL + PMC (21%), 6 PCL + PLC (14.2%), 7 PCL + ACL + PLC (16.7%) and 7 PCL + ACL + PMC (16.7%).

**Table 1:** Demographic data of patient’s included

Demographic data	
Follow-up time, years	9.6 ± 5.6 [2–28]
Age at surgery, years	31.4 ± 12.2 [13–59]
From trauma to surgery, years	3.3 ± 7.3 [0.1–35]
Sex, M/F	39/3
Limb, R/S	23/19
BMI, ≤ 25 / > 25	28/14
Diabetes, Y/N	4/38
Smoke, Y/N	14/28
Meniscal lesion, Y/N	10/32
Cartilage lesion, Y/N	7/35

### Clinical outcomes

A significant improvement in all PROMS was noted from pre- to post-surgery (range η_p_^2^ 0.21–0.43, *p* < 0.05) except for the Tegner score. No differences were noted between the post-op and the final follow-up (n.s.) (Table 2). The pre-injury Tegner score was never reached after surgery, but a significant improvement was noted between the pre-surgery and the final follow-up (η_p_^2^ = 0.67,* p* < 0.001) (Table 2). The RTW was achieved in the 95.2% (40/42) after 2.4 ± 1.9 months and RTS was achieved in 78.6% (33/42) after 6.7 ± 5.0 months. The higher number of surgeries were the significant negative predictors of PASS for the KOOS sub-scales Sport (*p* = 0.040) and Quality of Life (*p* = 0.046), while the presence of meniscal lesions was a significant negative predictor of PASS only for the KOOS sub-scale of Sport (*p* = 0.003).

**Table 2:** Clinical outcomes pre-injury, pre-surgery, post-surgery

Clinical outcomes						
Score	Pre-injury	Pre-surgery	Post-surgery	Final follow-up	η_p_^2^	*p* value
VAS		43.5 ± 26.3	28.1 ± 23.3^a^	20.9 ± 21.5^a^	0.43	< 0.001
Lysholm		73.0 ± 18.9	76.8 ± 14.3	82.3 ± 8.7	0.20	n.s
KOOS–ADL		57.7 ± 12.8	62.9 ± 11.9^a^	64.9 ± 11.7^a^	0.30	0.002
KOOS–Pain		72.5 ± 24.3	79.6 ± 18.0	83.5 ± 16.9^a^	0.21	0.019
KOOS–Function		78.0 ± 26.0	85.3 ± 18.8	89.5 ± 16.5^a^	0.21	0.017
KOOS–Sport		54.0 ± 32.6	61.8 ± 29.2	69.0 ± 27.0	0.22	0.015
KOOS–QoL		47.7 ± 31.3	57.8 ± 26.7	63.4 ± 26.2	0.28	0.004
Tegner	8 [0–10]	2.5 [0–10]	5 [0–10]	5 [0–10]^a,b^	0.67	< 0.001

^a^Difference from pre-surgery

^b^Difference from post-surgery

### Survivorship analysis

**Fig. 1:**
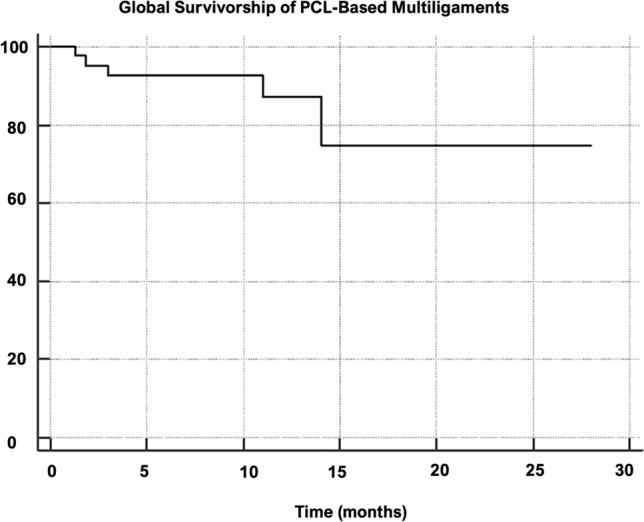
Global survivorship of PCL-based multiligaments

**Fig. 2:**
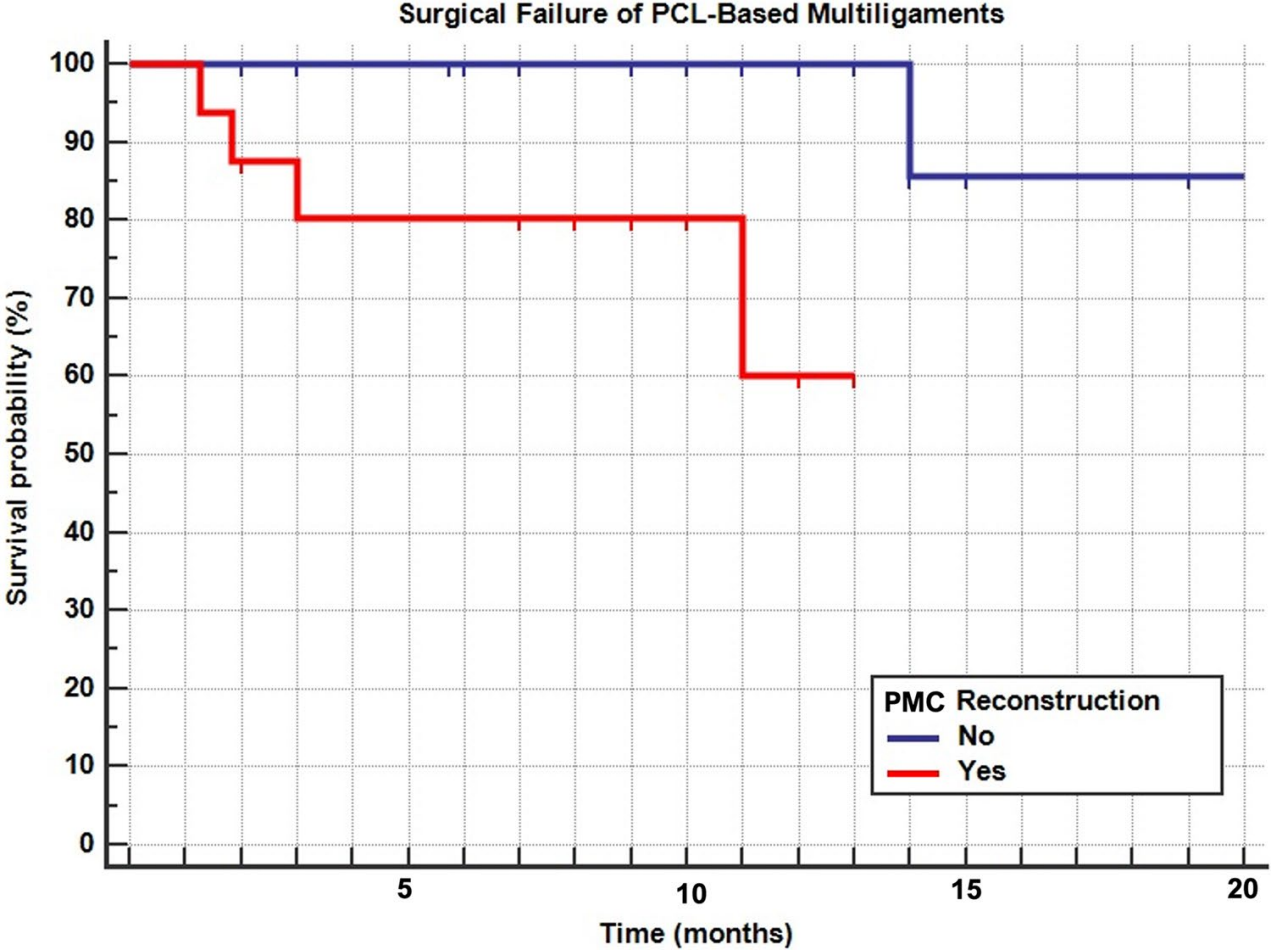
Surgical failure of PCL-based multiligaments

Six patients (14.3%) underwent reoperation and were considered as surgical failures (Table 3). The global survivorship was 95.2%, 92.6%, 87.1%, and 74.7% at 2, 5, 12, and 15 years, respectively (Fig. 1). The survivorship in patient undergoing PMC reconstruction surgery was significantly lower (*p* = 0.004; HR 7.1) compared to patients without a PMC lesion (Fig. 2). No survival differences were found between the ACL-PCL-PMC and the ACL-PCL-PLC (n.s.). No significant outcome interaction emerged from the multiple regression analysis (n.s.).

**Table 3:** Surgical failure according to the surgical intervention

Surgical failure according to the surgical intervention				
Surgery	Surgical failure		Total number of patients	% of surgical failure
	No	Yes		
PCL	11	0	11	0.0
ACL + PCL	1	1 (PCL)	2	50.0
PCL + PMC	8	1 (PCL)	9	11.1
PCL + PLC	5	1 (PCL)	6	16.7
PCL + ACL + PLC	7	0	7	0.0
PCL + ACL + PMC	4	3 (PCL/PMC/PCL + PMC)	7	42.9
Total	36	6	42	14.3

## Discussion

The most important finding of the present study was that good-to-excellent PROMS could be obtained and maintained at long-term follow-up after PCL-MLKI, with the higher number of surgeries and meniscal lesions as significant negative predictors of the PASS. Moreover, the presence of a PMC lesion significantly increases the risk of the PCL reconstruction failure.

Clinical outcomes after multiligament knee injury are difficult to be compared, due to the low incidence of the pathology, the heterogeneity of the lesions and the different possible treatments. What is somehow commonly accepted is that the surgical treatment of these conditions is the best solution. Ritcher et al., in fact, compared a group of patients which underwent surgical treatment of MLKI with patients which were treated only with rehabilitation. The last reported lower clinical outcomes in terms of knee function and return sport and to work [23]. Also, Wong et al. reported better outcomes in terms of IKDC and subjective instability for patients underwent operative treatment [25]. In the present series we noted that patients could be able to reach significantly better outcomes post-operatively and this result remained stable throughout the years. Interestingly the outcomes were significantly influenced by the meniscal status found at the time of surgery and the higher number of surgeries. In authors opinion these two factors are extremely correlated. It is well established that objective instability not only influence patient’s perception of the knee, but more importantly affects the meniscal and cartilage condition with a generally reported higher incidence of meniscal lesion in chronic ligaments deficiency scenarios [16]. Also, Moathse et al. in a prospective review of 303 patients reported about 40% of meniscal tears and a higher incidence of cartilage injury in chronic scenarios [13, 17]. Hence, this finding seems to suggest that an early and not staged surgical management of such injuries could be a better solution, even though this topic is still controversial [7, 15, 19–21]. Focusing on the return to previous activity we found different scenarios. The possibility to RTW was well achieved by the whole population with a rate of 95% after almost 2 months, as also found by Worley et al.[26] Regarding the RTS there was a significant improvement from the pre-operative to the post-operative Tegner level which remained stable till the last follow-up, even though none reached the pre-injury level. In authors opinion, these findings could be explained by the age of the population analyzed (30 years old average). This age range, in fact, usually need to rapidly return to work, but regarding the RTS, on the other hand, the low rate of return to the pre-injury level highlights the complexity and possibly life-changing burden of these injuries, since sport participation is perceived as an important aspect of the life, as demonstrated by a rate of RTS of almost 80%, even at a lower level. These findings are in line with other researchers [1, 4, 7, 17]. Moathse et al. [17] reported in the same age group similar rates of RTW and RTS as also Godin et al. which analyzed older adolescent population (17 years old average) reporting an improvement of the Tegner from pre- to post-operation, but also concluding that such injuries could lead to impaired function [7]. Also, in a recent systematic review Everhart et al. highlighted a high RTW rate (88.4%) but a low RTS at any level (53.6%) and even lower if considering high level of sports (22–33%) [4]. Another crucial aspect that influenced the RTS findings were the number of surgeries and meniscal lesions which negatively affected the Sport KOOS sub-scale. Meniscal tears are highly represented in MLKI [11], reported in almost the 30% of patients in the present series (Table 1), and their detrimental role is in line with King et al. findings, which reported significant lower clinical outcomes at 6 years follow-up in 121 patients with cartilage and combined meniscal tears [12].

These two aspects could have influenced the PASS from different sides. One is the need of a long rehabilitation process after every surgical intervention, usually from 6 to 12 months for a safe return to impact activities. From the other side the presence of meniscal lesions could have driven the patients to a more conservative behavior in terms of sport participations. Interestingly the sub-scale of sports and quality of life were influenced by the same factors, furtherly supporting the idea that the quality of life is somehow influenced by sport activity. To authors knowledge this is the first study which investigated the possible factors influencing the PASS in MLKI patients.

Focusing on the survivorship analysis, another notable finding of the present study is that patients who presented a PMC lesion were burdened by a sevenfold increased risk of failure of the PCL reconstruction. Overall, more than 92% of reconstructed knees still were revision-free at 5 years follow-up, and only after the first decades these patients underwent a revision of at least one ligament previously reconstructed, with a relevant drop at 15 years. Different authors have highlighted a higher failure rate when repair of the cruciate and collateral ligaments is performed, thus suggesting for anatomic reconstruction of the torn ligaments [7, 12, 14]. In the present study it is not possible to drive such conclusion since the cohort is variegated and not always so-called anatomic reconstructions have been performed. However, its authors opinion, and these findings seems support this, that in MLKI surgeries scenarios also repairs and non-anatomic reconstruction could drive to excellent outcomes as also suggested by Hanley et al. [9]. Interestingly in the present series there was a significantly lower survivorship for medial-sided injuries. Also, King et al. [12] in a cohort of 56 patients at 6-year follow-up reported poorer outcomes in patients underwent medial sided injuries. This could be explained by the higher incidence of such injuries in MLKI scenarios [7] and by the anatomy of the PMC itself. In fact the wide tibial insertion of the superficial MCL, the deep MCL blended with the capsule and thin width and different orientation of the POL makes its repair and reconstruction difficult to be performed with a usually not avoidable grade of residual laxity which could overload the PCL reconstructed and so leading to its failure [3, 18].

The present study has some limitations. First the relatively low number of patients included, in fact, some data could have reached the statistical significance if analyzed in a wider cohort. Second, the difficult generalizability of these findings is difficult. Third the retrospective nature of the study. However, the present case-series presented the results of number of subjects in line with the wideness of other cohort studies on the same topic. In addition, to authors knowledge, this is the first study that indented to evaluate the possible factors influencing the PASS in such injuries, performed a survival analysis with the risk factors associated and presented results at such long follow-up (average 11.9 years). Moreover, given the variety and rarity of presentation of these injuries, designing high level of evidence trials is difficult, and the contribution of not-high-level-evidence data is important. Undoubtedly, the need of multicentric study of dramatic usefulness as suggested by Hankins et al. [8]. Despite these limitations the authors believes that the clinical relevance of this study derives from the deepening of the knowledge into the MLKI; in particular, regarding the need of further surgical major procedures which can help the surgeon to set patient’s expectations in these complex scenarios.

The clinical relevance of this study derives from the deepening of the knowledge into the MLKI; from one side, regarding the risk of further ligament revision surgery in case of medial-sided injuries and from the other side, to set patient’s expectations regarding long-term outcomes and return-to-sport rate which even if high was never at pre-injury level.

## Conclusions

Good-to-excellent PROMS could be obtained and maintained at long-term follow-up after surgery, with the higher number of surgeries and meniscal lesions as significant negative predictors of the PASS. Moreover, the presence of a PMC lesion significantly increases the risk of the PCL reconstruction failure.

## Data Availability

The authors declare that the data supporting the findings of this study are available within the article, additional data are available from the corresponding author upon request.
